# [Retracted] Resistin increases the expression of NOD2 in mouse monocytes

**DOI:** 10.3892/etm.2026.13187

**Published:** 2026-05-18

**Authors:** Yi Ren, Taomei Wan, Zhicai Zuo, Hengmin Cui, Xi Peng, Jing Fang, Junliang Deng, Yanchun Hu, Shuming Yu, Liuhong Shen, Xiaoping Ma, Ya Wang, Zhihua Ren

Exp Ther Med 13:2523–2528, 2017; DOI: 10.3892/etm.2017.4288

Following the publication of the above paper, a concerned reader drew the Editor's attention to the fact that, regarding the western blot data shown in Fig. 3 in p. 2527, the bands shown for NOD2 (the NOD2 siRNA, 24 h experiment) in Fig. 3A and RIP2 (the band in the first lane on the left) in Fig. 3B were remarkably similar. In addition, after having conducted an independent investigation of this paper in the Editorial Office, a pair of the β-actin bands featured in Fig. 3A also were found to be highly similar.

Based on a consideration of the above points, the Editor of *Experimental and Therapeutic Medicine* has determined that the paper should be retracted from the Journal on account of a lack of confidence in the presented data. The authors did reply to our query concerning the potentially anomalous aspects of the western blots in Fig. 3A and B, and considered that the data were different to the best of their knowledge, although owing to the time that has elapsed since this paper was published, they no longer had access to the original data underlying these experiments. The Editor regrets any inconvenience that has been caused to the readership of the Journal.

